# The chain mediating role of psychological resilience and neuroticism between intolerance of uncertainty and perceived stress among medical university students in Southwest China

**DOI:** 10.1186/s12888-023-05345-z

**Published:** 2023-11-21

**Authors:** Xiaoxiao Xu, Xiaofan Yan, Qianhui Zhang, Chen Xu, Min Li

**Affiliations:** 1 Department of Military Psychology, Faculty of Medical Psychology, Army Medical University (Third Military Medical University), Chongqing, 400038 China; 2 Department of Basic Psychology, Faculty of Medical Psychology, Army Medical University (Third Military Medical University), Chongqing, 400038 China; 3 Department of Foreign Languages, College of Basic Medical Sciences, Army Medical University (Third Military Medical University), Chongqing, 400038 China

**Keywords:** Medical university students, Intolerance of uncertainty, Perceived stress, Psychological resilience, Neuroticism

## Abstract

**Background:**

Medical university students are confronted with unprecedented uncertainty and stress compared with their peers. Research has explored the effect of intolerance of uncertainty on perceived stress, but little attention was paid to investigate the mediating mechanisms behind this relationship, especially among medical university students. The aim of this study was to examine whether psychological resilience and neuroticism played a mediating role between medical university students’ intolerance of uncertainty and perceived stress.

**Methods:**

A total of 717 medical university students from Chongqing in Southwest China were recruited to participate in our study and completed demographic information, Intolerance of Uncertainty Scale Short Version (IUS-12), Chinese Version of Perceived Stress Scale (CPSS), Connor-Davidson Resilience Scale-10 (CD-RISC-10) and Eysenck Personality Questionnaire (EPQ).

**Results:**

(1) Significant correlations between intolerance of uncertainty, perceived stress, psychological resilience and neuroticism were found. (2) Intolerance of uncertainty affected medical university students’ perceived stress via three paths: the mediating effect of psychological resilience, the mediating effect of neuroticism, and the chain mediating effect of both psychological resilience and neuroticism.

**Conclusions:**

Intolerance of uncertainty could directly affect the perceived stress of medical university students, and also affected perceived stress through the mediating roles of psychological resilience and neuroticism, as well as through the chain mediating role of these two variables.

## Introduction

Perceived stress is defined as a situation that individuals evaluate as threatening or otherwise demanding and for which they lack sufficient resources to address [1]. Exposure to low level of stress may benefit individuals both physiological and psychological health. However, severe or chronic stress is found to associate with negative health consequences, such as depression symptoms, suicide ideation, poorer sleep quality, long-term sickness and even mental disorders [2]. As the successors of medical workers, medical university students have been reported experiencing a higher level of stress compared with their peers, such as the stress of schooling, academic pressure and the stress of clinical practice [3]. With the demand for medical education strives to cultivate competent and well-rounded physicians, promoting medical university students’ mental health has received increased attention [4].

### Relationship between intolerance of uncertainty and perceived stress

Intolerance of uncertainty (IU) is a cognitive bias that perceives, interprets and reacts to uncertain situations or events, which affects individuals’ cognition, emotion and behavioral responses [5]. Specifically, individuals with higher IU may believe that uncertainty is stressful and frustrating, and perceive uncertainty as negative and threatening. Furthermore, they may try to control their future, avoid and inhibit uncertainties [5]. A growing number of evidence suggests that IU may be an important transdiagnostic maintaining factor underlying various kinds of psychological disorders [6, 7]. Psychopathological theories of IU consider that the tendency to feel distressed caused by uncertainty, as well as the behavioral and psychological attempts to decrease the uncertainty, may be the main symptoms of a variety of mental disorders [6, 7]. The cognitive vulnerability model considers that IU is a crucial high comorbidity factor observed across emotional disorders, including generalized anxiety disorder, depressive disorder [6]. Therefore, the extent to which individuals are able to tolerate uncertainty is an important characteristic that affects mental health.

Uncertainty is an inevitable part of medical practice whether from increasingly complex disease or conflicting clinical information. Tolerate uncertainty of doctors is viewed as a key competency by regulating bodies in the US, European Union, Canada and Australia [8]. There is growing evidence indicates that the incidence of stress, burnout and mental disorders among doctors around the world is fairly high. Compared with age matched peers who are not medical students, higher levels of depression, anxiety and perceived stress among medical students have been confirmed [4]. Several systematic reviews have proposed positive relationships between IU in clinical practice and series of mental health outcomes, for example, psychological distress, which may reduce psychological well-being [9, 10]. Hancock and Mattick found that there seemed to be a correlation between intolerance of ambiguity or uncertainty and the decreased psychological well-being (stress, burnout or a mental health disorder) among both medical students and doctors [9]. Previous studies have also found that higher IU was associated with higher stress, anxiety and depressive symptoms [11], poor decision-making ability, decreased coping skills, low level of motivation, avoidance of ambiguity, and declined academic performance [12]. Therefore, we hypothesize that IU is positively associated with perceived stress.

### The potential mediating effect of psychological resilience

Psychological resilience involves to individuals’ ability of coping with stress and developing adaptability when faced with adversities, which helps people effectively deal with stress, anxiety, depression, trauma and maintain mental health [13]. In today’s fast-changing world, it’s necessary for everyone to cultivate high level of psychological resilience [14]. Rahimi et al. [15] indicated that medical students with higher levels of psychological resilience exhibited lower levels of perceived stress, and psychological resilience could play a role in buffering perceived stress when faced daily or chronic stressors, including exams, class timetable or an illness. Garcia-Leon et al. [16] evaluated whether resilience was related to perceived stress, chronic stress and life events among healthy adults, and results showed a higher level of perceived stress in the low resilience group when compared with the high resilience group. Highly resilient adults may believe that they would be better able to confront with stress and less overwhelmed by stressors. In addition, a few of studies have explored the mediating role of psychological resilience in perceived stress. For example, Sarrionandia et al. [17] examined the impacts of emotional intelligence and resilience on students’ perceived stress, and revealed that emotional intelligence acted as a negative predictor of perceived stress through the mediating variable of resilience for the American and Basque students. However, there was limited literature on the relationship between IU and psychological resilience. Lee et al. [18] showed that IU was negatively related to resilience in nursing university students. In particular, higher positive tendencies led to lower level of IU. Wang et al. [19] found that high level of IU had adverse impacts on mental burden during the COVID-19 pandemic, while individuals with high level of resilience could weak this impacts. The combined observations of the correlations between IU, psychological resilience and perceived stress suggested that psychological resilience might mediate the relationship between IU and perceived stress. Therefore, we infer that the impact of IU on perceived stress is mediated by resilience.

### The potential mediating effect of neuroticism

Personality acts a crucial role in the occurrence and development of mental health problems. Neuroticism is defined as the tendency to experience frequent and intense negative emotions, cognitive and maladaptive behaviors in response to various sources of stress [20–22]. Previous studies have confirmed that personality differences regulated stress process [23], and the most consistent finding was that neuroticism was positively related with higher stress appraisals and greater reactivity to stressful events [23, 24]. Mohitedini et al. [25] assessed participants’ level of neuroticism, and then during a Trier Social Stress Test, individuals that scored high in neuroticism experienced more stress than those scored low in neuroticism. Greater perceived stress was also reported during the COVID-19 pandemic for individuals with high neuroticism in France [26], Slovenia [27], Italy [28] and Germany [29]. Higher neuroticism predicted higher levels of hair cortisol, cortisone and subjective stress [30]. The integrative model of IU stated that personality may buffer or intensify responses to perceived uncertainties [31]. Previous literature suggested that IU was an individual difference variable which was conceptualized as the result of personality, especially neuroticism, and IU shared a robust association with neuroticism [32]. According to the Big Five personality theory, individuals with high neuroticism were prone to unrealistic ideas and excessive demands. When individual with higher neuroticism encountered uncertain situations, they might exhibit IU and suffer from mental disorders [33]. Therefore, we assume that the impact of IU on perceived stress is mediated by neuroticism based on theoretical and empirical evidence.

### The chain mediating effect of psychological resilience and neuroticism

As we assumed above, both the psychological resilience and neuroticism may play mediating roles of between IU and perceived stress. Interestingly, however, what is the relationship between psychological resilience and neuroticism when they are both considered to be the mediating factors of IU in perceived stress? Which plays a more important mediating role than the other? A previous meta-analysis revealed a stronger negative relationship between psychological resilience and neuroticism [34]. Psychological resilience affects individuals’ neuroticism, and low psychological resilience individuals have significantly higher neuroticism than high resilience individuals [35, 36]. Besides the above two single mediating role, there may be a chain mediating role affecting the relationship between IU and perceived stress.

### The current study

Therefore, we constructed a theoretical hypothesis model and proposed the following four hypotheses: (1) IU could positively predict the perceived stress of medical university students. (2) IU could indirectly predict the perceived stress of medical university students via the mediating role of psychological resilience. (3) IU could indirectly predict the perceived stress of medical university students through the mediating role of neuroticism. (4) IU could indirectly predict the perceived stress of medical university students through the mediating role of psychological resilience and neuroticism.

## Methods

### Participants

This current research has been reviewed and approved by the Medical Ethics Committee of the Army Medical University (No.2020-023-03). The authors of this study were the data collectors who received training to conduct the survey and response to questions from the subjects. After reading the informed consent, participants could choose whether continue to participate in this study by their own will or exit the study at any time. At the same time, researchers guaranteed that they didn’t disclosed any contents of this study and personal information. A total of 717 medical university students participated our study. Females were 253 students and males were 464 students. Ages of them ranged from 17 to 25 years old (M = 19.76, SD = 1.23). All the participants distributed in different grades, of which 104 (14.50%) students were in their first year of university, 321 (44.77%) students were in their second year of university, 235 (32.78%) students were in the third year of university, 49 (6.83%) students were in the fourth year of university and 8 (1.12%) students were in the last year of university.

### Measures

#### Intolerance of uncertainty scale short version (IUS-12)

IUS-12 was typically applied to evaluate individuals’ responses to uncertainty, the future and ambiguous situations. This scale consisted of 12 items and utilized a 5-point Likert scale ranging from 1 (not at all characteristic of me) to 5 (very characteristic of me), resulting possible total scores from 12 to 60 [37]. The higher overall scores, the higher IU. The Cronbach’s Alpha of IUS-12 in this current study was 0.84.

#### Chinese version of perceived stress scale (CPSS)

Perceived stress was evaluated by using the Chinese version of the 14-item Perceived Stress Scale (CPSS) [38]. Items were rated from 0 (never) to 4 (very often), and seven of them (4, 5, 6, 7, 9, 10, 13) were reverse-scored. The total scores were the sum of all items with reverse coding of relevant items, and it ranged from 0 to 56. The higher total scores, the higher perceived stress. The Cronbach’s Alpha of CPSS in this current study was 0.89.

#### Connor-Davidson Resilience Scale-10 (CD-RISC-10)

The psychological resilience of medical university students was assessed by the 10-item Connor-Davidson resilience scale (CD-RISC-10) [39], which was revised based on the 25-item CD-RISC [13]. The responses of each item were scored on a 5-point Likert scale, ranging from 0 (not true at all), 1 (rarely true), 2 (sometimes true), 3 (often true), and 4 (true nearly all the time). The total score could range from 0 to 40 with higher scores indicating greater resilience of participants. The Cronbach’s Alpha of this whole scale in this current study was 0.89.

#### Eysenck Personality Questionnaire (EPQ)

The complete version of the EPQ scale had been verified in the Chinese population with acceptable reliability and validity, and the neuroticism subscale of the EPQ was used to assess individuals’ personality traits [40]. Neuroticism personality was an emotional trait, which was manifested in the tendency of rapid arousal and slow inhibition of emotion when stimulated. It comprised 12 items, to which participants were asked to provide a yes (1) or no (0) answer. The total original score ranged from 0 to 12, with a higher score indicating a higher level of neuroticism. The Cronbach’s Alpha of Neuroticism in this current study was 0.89.

### Statistical analysis

The analysis of current study was conducted using the SPSS 22.0 software. We first conducted descriptive statistics on demographic variables (age, gender and grade) and four scales scores (IU, perceived stress, psychological resilience and neuroticism), and then standardized the data of the four scales scores. The relationships between IU, perceived stress, psychological resilience, and neuroticism were explored by Person correlation analysis. We used the skewness and kurtosis tests to comprehensively examine the distribution normality for the four scales scores. In order to investigate the mediating roles of psychological resilience and neuroticism between IU and perceived stress, the SPSS PROCESS macro 3.3 software [41] was used. The mediating effects of psychological resilience and neuroticism were tested by applying model 6 in PROCESS. A bias-corrected bootstrapping procedure was used to calculate indirect effects. If the 95% confidence interval (CI) did not include 0, it meant that the mediation effect was significant [42]. Gender and age were included as covariates in the models.

## Results

### Common method biases tests

In order to inspect the common method biases caused by self-reported scales, Harman’s single factor test was conducted [43]. The first factor accounted for 38.36% of the total variation, which was lower than the value of 40% raised by Podsakoff et al. [44]. It indicated that the common method bias was unlikely to confuse the interpretation of data analysis results [42].

### Descriptive analysis and correlations between variables

The basic descriptive data for IU, perceived stress, psychological resilience and neuroticism were shown in Table 1. Specifically, the mean total scores for IU were 33.64 ± 7.58, the mean total scores for perceived stress were 35.94 ± 8.79, the mean total scores for psychological resilience were 25.00 ± 6.39, and the mean total scores for neuroticism were 49.16 ± 12.82. And we conducted the skewness and kurtosis tests to comprehensively examine the distribution normality for above variables. For sample size greater than 300, the approximate normal distribution was defined for variables with absolute values of skewness below 3 and kurtosis below 8 [45]. As shown in Table 1, the skewness values of each variable ranged from − 0.13 to 0.48 and the kurtosis values ranged from − 0.99 to 0.04, indicative of normal distribution. Pearson correlation analyses were used to test the correlations of all the variables. As shown in Table 1, all the variables were significantly correlated with each other. IU was positively correlated with perceived stress (*r* = 0.62, *P* < 0.01) and neuroticism (*r* = 0.60, *P* < 0.01), and negatively related with psychological resilience (*r* = -0.52, *P* < 0.01). Perceived stress was positively correlated with neuroticism (*r* = 0.71, *P* < 0.01) and negatively correlated with psychological resilience (*r* = -0.80, *P* < 0.01). Psychological resilience was negatively correlated with neuroticism (*r* = -0.62, *P* < 0.01).

**Table 1:** Means, standard deviations, skewness, kurtosis and correlations for study variables

Variables	Mean	SD	Skewness	Kurtosis	1	2	3	4
1.Intolerance of uncertainty	33.64	7.58	0.17	-0.28	1			
2.Perceived stress	35.94	8.79	0.26	-0.26	0.62**	1		
3.Psychological resilience	25.00	6.39	-0.13	0.04	-0.52**	-0.80**	1	
4.Neuroticism	49.16	12.82	0.48	-0.99	0.60**	0.71**	-0.62**	1

N = 717

All tests were two-tailed. This table shows the general means, standard deviations, skewness, kurtosis and correlations of the four major variables. ** indicates a significant correlation between the variables. ** *P* < 0.01

### Psychological resilience and neuroticism: the chain mediating effects analyses

**Fig. 1:**
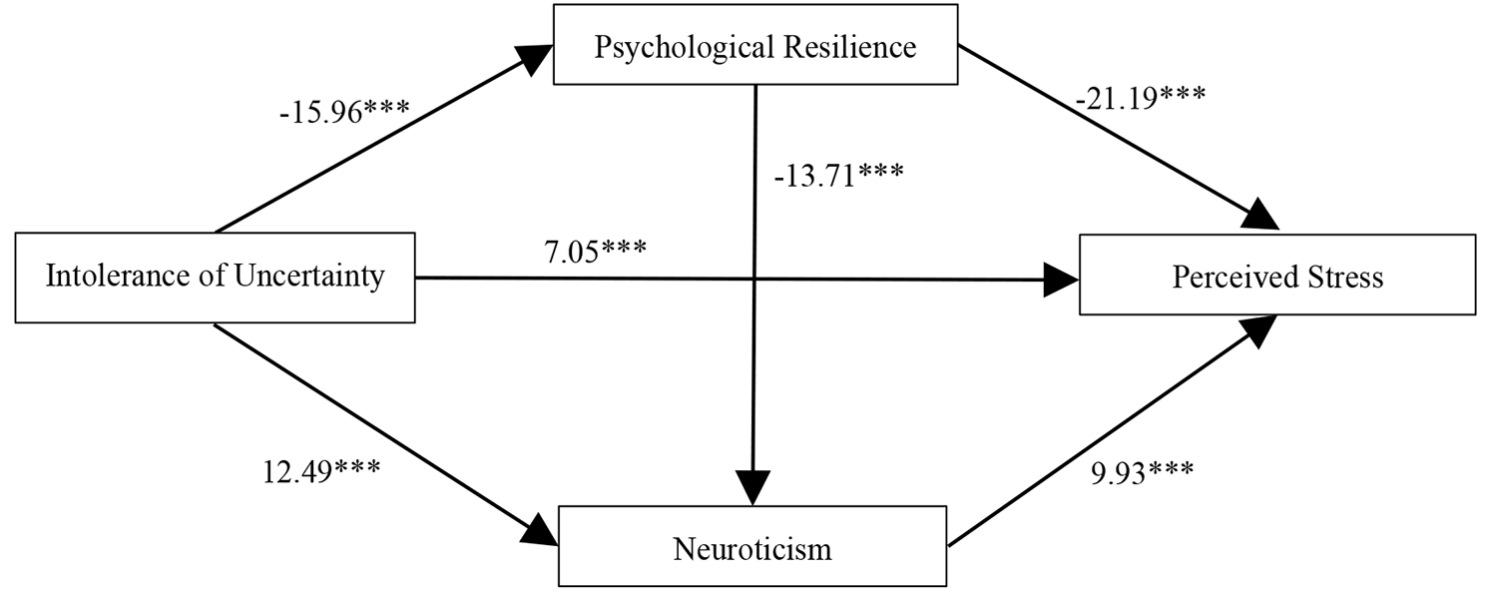
Chain mediation model of IU, perceived stress, psychological resilience and neuroticism. *** *P* < 0.001

There were significant correlations between IU, perceived stress, psychological resilience and neuroticism, which met the statistical requirements for further mediating effect analysis of between IU and perceived stress [46]. We used the model 6 in SPSS 22.0 compiled by Hayes [41] to analyze the mediating roles of psychological resilience and neuroticism in the relationship between IU and perceived stress. The results of regression analyses were listed in Table 2. After controlling for gender and age, IU were negatively associated with psychological resilience (*β* = -0.51, *P* < 0.001) and positively associated with neuroticism (*β* = 0.38, *P* < 0.001) and perceived stress (*β* = 0.17, *P* < 0.001). Psychological resilience could negatively predict neuroticism (*β* = -0.42, *P* < 0.001) and perceived stress (*β* = -0.54, *P* < 0.001). Besides, neuroticism was a significant positive predictor of perceived stress (*β* = 0.27, *P* < 0.001). Figure 1 represented the model plot after the testing.

**Table 2:** Regression analyses of relationships between variables in the mediation model

Dependent variable	Independent variable	*β*	*SE*	*t*	*R* ^*2*^	*F*
Psychological resilience	Gender	-0.06	0.03	-2.03*	0.28	90.85***
	Age	-0.002	0.03	-0.08		
	Intolerance of uncertainty	-0.51	0.03	-15.96***		
Neuroticism	Gender	-0.01	0.02	-0.50	0.50	181.30***
	Age	0.05	0.02	2.20*		
	Intolerance of uncertainty	0.38	0.03	12.49***		
	Psychological resilience	-0.42	0.03	-13.71***		
Perceived stress	Gender	-0.004	0.02	-0.22	0.74	396.83***
	Age	-0.007	0.02	-0.38		
	Intolerance of uncertainty	0.17	0.02	7.05***		
	Psychological resilience	-0.54	0.03	-21.19***		
	Neuroticism	0.27	0.03	9.93***		

N = 717

All variables in the model have been standardized. This table presents the results of the multiple hierarchical regression analysis of intolerance of uncertainty, psychological resilience, neuroticism and perceived stress

* *P* < 0.05, *** *P* < 0.001

Table 3 showed the mediating roles of psychological resilience and neuroticism in the correlation between IU and perceived stress. Figure 1 was a chain mediating model between IU and perceived stress. Both the Table 3 and Fig. 1 suggested that psychological resilience and neuroticism played significant mediating roles in between IU and perceived stress. The total effect of IU on perceived stress was 0.62, the direct effect of IU on perceived stress was 0.18 and the total standardized mediating effect value was 0.44. The proportion of the total standardized mediating effect to the total effect was 70.97%. The mediating effect consisted three pathways. Path 1: IU → psychological resilience → perceived stress (0.28). Path 2: IU → neuroticism → perceived stress (0.10). Path 3: IU → psychological resilience → neuroticism → perceived stress (0.06). The three indirect effects of path 1, path 2, and path 3 accounted for 45.16%%, 16.13% and 9.68%, respectively. All of three indirect effects met the significant level, as the 95% confidence interval of these indirect effects did not contain the 0 value. Comparisons showed that the bootstrap 95% confidence interval for the difference between indirect effects 1 and 2 and between indirect 1 and 3 didn’t contain 0 value, indicating that the path 1 was significantly different from path 2 and 3. When the same compared method was conducted between 2 and 3, the bootstrap 95% confidence interval for the difference contained a 0 value which means that there was no significant difference between them. These results indicated that IU could indirectly predict perceived stress not only through the single mediating effect of psychological resilience and neuroticism, but also through the chain mediating role of psychological resilience and neuroticism.

**Table 3:** Psychological resilience and neuroticism in the mediation effect analysis

	Effect value	Boot SE	Boot LLCI	Boot ULCI	Effect proportion
Total effect	0.62	0.03	0.00	0.56	
Direct effect	0.18	0.03	0.00	0.13	29.03%
Total indirect effect	0.44	0.03	0.38	0.51	70.97%
Indirect effect 1	0.28	0.03	0.22	0.33	45.16%
Indirect effect 2	0.10	0.02	0.08	0.14	16.13%
Indirect effect 3	0.06	0.01	0.04	0.08	9.68%
Compare 1	0.17	0.03	0.11	0.24	
Compare 2	0.22	0.03	0.17	0.27	
Compare 3	0.05	0.01	0.02	0.07	

N = 717

All variables in the model were standardized. The direct and indirect effects of IU on perceived stress are shown in this table. Boot SE, Boot LLCI, and Boot ULCI refer, respectively, to the standard error and the lower and upper limits of the 95% confidence interval of the effects estimated by the percentile bootstrap method with deviation correction. Indirect effect 1: IU → psychological resilience → perceived stress; Indirect effect 2: IU→ neuroticism → perceived stress; Indirect effect 3: IU → psychological resilience → neuroticism → perceived stress. Compare 1: indirect effect 1-indirect effect 2; Compare 2: indirect effect 1-indirect effect 3; Compare 3: indirect effect 2-indirect effect 3

## Discussion

Our current research established a chain mediation model to explore the relationship between IU and perceived stress among medical university students in Southwest China. We confirmed a positive relationship between IU and perceived stress, which partially mediated by both psychological resilience and neuroticism through the three pathways: psychological resilience, neuroticism, and psychological resilience→neuroticism. These will help us deeply understand the relationship between IU and perceived stress, and provide guidance for effectively enhancing the tolerate uncertainty and alleviating perceived stress of medical university students.

### Effects of IU on medical university students’ perceived stress

Firstly, our results showed that IU could positively predict higher levels of medical university students’ perceived stress, which was consistent with previous studies. Abundant of studies in the context of both normal time and COVID-19 pandemic indicated that higher IU was positively correlated with perceived stress [47–51]. For example, Weist [51] indicated that IU could significantly predict students’ level of depression, anxiety and stress. Furthermore, our finding reinforced the role of IU as a transdiagnostic risk factor associated with adverse mental health outcomes. IU has been considered as a specific risk factor or cognitive vulnerability in the development and maintenance of anxiety symptoms (e.g., worrying and catastrophizing) [52] and generalized anxiety disorder [53].

As the reserve talents of medical talent team, medical students need to face many unknowns and uncertainties during their study and future employment. Exploring the relationship between IU and perceived stress will help to provide intervention targets. Cultivating medical students’ tolerance of uncertainty and enhancing their psychological resources to cope with uncertainty are not only beneficial for their mental health, but also for their future career choices and development. While IU negatively impacted medical diagnosis, decision-making and doctor-patient communication, tolerance of uncertainty could affect physicians’ attitude towards patients and future career directions during clinical internships [54]. Therefore, understanding the impacts of IU and the correlation between IU and perceived stress has practical significance. Enough attention should be paid to medical university students’ IU and targeted coping strategies should be taken, thereby reducing their perceived stress.

### The mediating role of psychological resilience between IU and perceived stress

Our result indicated that psychological resilience had a mediating effect on the relationship between IU and perceived stress, which was in accordance with prior research [17, 19]. As mentioned above, Wang et al. [19] found that high IU negatively impacted individuals’ mental burden during the period of COVID-19 pandemic and high psychological resilience weakened the adverse impact. In addition, Sarrionandia et al. [17] revealed the mediating role of psychological resilience in the negative predictor relationship between emotional intelligence and perceived stress among the American and Basque students. It has also been proven that resilient coping was an important moderator, which altered the strength of the association between IU and distress reactions [55]. These evidences indicated that resilience was a protective factor for the physical and mental health when individuals experienced or faced adversities. When individuals with high levels of IU faced uncertain situations, resilience may reduce perceived stress and play a buffering role in combating mental vulnerability and emotional distress. It meant that the positive correlation between IU and perceived stress would be weakened if individuals responded with a high level of psychological resilience. Individuals may actively seek information or find solutions to handle all perceived uncertainties. Resilience was likely to be a protective factor that could enhance individuals’ ability of tolerance to distress, as well as a component that contributes to constructive appraisals of the uncertainty. Therefore, enough attention should be paid to improve the resilience of medical students in order to reduce the possibility of emotional distress when facing uncertain situations or events.

### The mediating role of neuroticism between IU and perceived stress

Consistent with previous studies, the present study also showed that neuroticism mediated the relationship between IU and perceived stress. Individuals with higher level of neuroticism often experienced higher perceived stress when exposed to the same level of uncertainty situations. During the past three years, the COVID-19 pandemic brought huge healthy, economic and social uncertainties, which caused great fear among people globally. Yang et al. [56] showed that fear of COVID-19 was positively correlated with perceived stress and neuroticism, and neuroticism mediated the relationship between fear of COVID-19 and perceived stress. Our finding reinforced that neuroticism was associated with stronger emotional responses, and individuals who scored high in neuroticism tend to be emotionally unstable and feel more psychological pressure. Therefore, in the selection of medical staff for important positions and special departments, lower neuroticism individuals would be screened as the potential candidates by early evaluation of personality characteristics.

### The chain mediating effect of psychological resilience and neuroticism between IU and perceived stress

In addition to the two mediating roles, we also found a chain mediating effect of psychological resilience and neuroticism on the relationship between IU and perceived stress. Namely, IU may lead to the increase of neuroticism by decreasing the psychological resilience, ultimately increasing individuals’ perceived stress. Li et al. [57] examined a set of hypothesized pathways using perceived stress, neuroticism and psychological inflexibility to predict depressive symptoms among Chinese new fathers. Neuroticism and psychological inflexibility played a chain mediating role in the relationship between perceived stress and depression. Psychological flexibility has been established as a cornerstone of mental and physical health, and is also an important factor for promoting psychological resilience [58]. Hence, it was likely that there was a chain mediating role of psychological resilience and neuroticism between IU and perceived stress. IU would exacerbate the adverse impact on individuals’ perceived stress by decreasing psychological resilience and increasing neuroticism.

### Limitations and implications

In summary, we explored the underlying mechanisms between IU and perceived stress among medical university students in Southwest China. These findings demonstrated that IU affected medical university students’ perceived stress through three different pathways: the single mediating role of psychological resilience, the single mediating role of neuroticism and the chain mediating role of both psychological resilience and neuroticism. Meanwhile, it was worth noting that there were several research limitations. Firstly, our data were self-reported and relevant essentially. Evidence from experimental and intervention research was very necessary and it would provide more abundant evidence for causal relationships. In addition, future work should use a longitudinal design to track the long-term impact of IU.

Despite the limitations mentioned above, our findings have significant theoretical and practical implications. In terms of theoretical significance, investigating the relationships between medical students’ IU, perceived stress, psychological resilience and neuroticism helps to clarify the mediating mechanism behind the impact of IU on perceived stress. For the practical significance, this study provides constructive psychological training pathways for medical educators, which may relieve the emotional distress and stress response of medical students when facing uncertain situations or events. According to the indirect effects of psychological resilience (45.16%) and neuroticism (16.13%) as shown in Table 3, both psychological resilience and neuroticism play crucial roles in the effects of IU on perceived stress. On the one hand, psychological resilience training can be provided for medical students in order to enhance their mental coping ability and alleviate their perceived stress in uncertain situations. On the other hand, emotional stabilization intervention trainings (e.g., mental psychological stabilization, emotional regulation, stress management) could also be provided for increasing medical students’ emotional stability, tolerance of uncertainty and promoting their mental health.

## Data Availability

Data is available on reasonable request from the corresponding author.
